# Cerebrospinal Fluid Biomarkers, Brain Structural and Cognitive Performances Between Normotensive and Hypertensive Controlled, Uncontrolled and Untreated 70-Year-Old Adults

**DOI:** 10.3389/fnagi.2021.777475

**Published:** 2022-01-12

**Authors:** Atef Badji, Joana B. Pereira, Sara Shams, Johan Skoog, Anna Marseglia, Konstantinos Poulakis, Lina Rydén, Kaj Blennow, Henrik Zetterberg, Silke Kern, Anna Zettergren, Lars-Olof Wahlund, Hélène Girouard, Ingmar Skoog, Eric Westman

**Affiliations:** ^1^NeuroPoly Lab, Institute of Biomedical Engineering, Polytechnique Montréal, Montréal, QC, Canada; ^2^Department of Neurosciences, Faculty of Medicine, Université de Montréal, Montréal, QC, Canada; ^3^Division of Clinical Geriatrics, Centre for Alzheimer Research, Department of Neurobiology, Care Sciences, and Society, Karolinska Institutet, Stockholm, Sweden; ^4^Department of Radiology, Karolinska University Hospital, Stockholm, Sweden; ^5^Department of Clinical Neuroscience, Karolinska Institutet, Stockholm, Sweden; ^6^Department of Radiology, Stanford Medicine, Stanford, CA, United States; ^7^Department of Psychiatry and Neurochemistry, Institute of Neuroscience and Physiology, Sahlgrenska Academy, Centre for Ageing and Health (AgeCap) at the University of Gothenburg, Gothenburg, Sweden; ^8^Clinical Neurochemistry Laboratory, Sahlgrenska University Hospital, Mölndal, Sweden; ^9^Department of Neurodegenerative Disease, UCL Institute of Neurology, London, United Kingdom; ^10^UK Dementia Research Institute at UCL, Mölndal, Sweden; ^11^Hong Kong Center for Neurodegenerative Diseases, Clear Water Bay, Hong Kong SAR, China; ^12^Department of Pharmacology and Physiology, Faculty of Medicine, Université de Montréal, Montréal, QC, Canada; ^13^Groupe de Recherche sur le Systéme Nerveux Central (GRSNC), Université de Montréal, Montréal, QC, Canada; ^14^Centre Interdisciplinaire de Recherche sur le Cerveau et l’Apprentissage (CIRCA), Université de Montréal, Montréal, QC, Canada; ^15^Centre de Recherche de l’Institut Universitaire de Gériatrie de Montréal (CRIUGM), Montréal, QC, Canada

**Keywords:** CSF (cerebrospinal fluid), hypertension, brain, MRI, white matter, aging

## Abstract

**Background:** Hypertension is an important risk factor for Alzheimer’s disease (AD). The pathophysiological mechanisms underlying the relationship between AD and hypertension are not fully understood, but they most likely involve microvascular dysfunction and cerebrovascular pathology. Although previous studies have assessed the impact of hypertension on different markers of brain integrity, no study has yet provided a comprehensive comparison of cerebrospinal fluid (CSF) biomarkers and structural brain differences between normotensive and hypertensive groups in a single and large cohort of older adults in relationship to cognitive performances.

**Objective:** The aim of the present work was to investigate the differences in cognitive performances, CSF biomarkers and magnetic resonance imaging (MRI) of brain structure between normotensive, controlled hypertensive, uncontrolled hypertensive, and untreated hypertensive older adults from the Gothenburg H70 Birth Cohort Studies.

**Methods:** As an indicator of vascular brain pathology, we measured white matter hyperintensities (WMHs), lacunes, cerebral microbleeds, enlarged perivascular space (epvs), and fractional anisotropy (FA). To assess markers of AD pathology/neurodegeneration, we measured hippocampal volume, temporal cortical thickness on MRI, and amyloid-β_42_, phosphorylated tau, and neurofilament light protein (NfL) in cerebrospinal fluid. Various neuropsychological tests were used to assess performances in memory, attention/processing speed, executive function, verbal fluency, and visuospatial abilities.

**Results:** We found more white matter pathology in hypertensive compared to normotensive participants, with the highest vascular burden in uncontrolled participants (e.g., lower FA, more WMHs, and epvs). No significant difference was found in any MRI or CSF markers of AD pathology/neurodegeneration when comparing normotensive and hypertensive participants, nor among hypertensive groups. No significant difference was found in most cognitive functions between groups.

**Conclusion:** Our results suggest that good blood pressure control may help prevent cerebrovascular pathology. In addition, hypertension may contribute to cognitive decline through its effect on cerebrovascular pathology rather than AD-related pathology. These findings suggest that hypertension is associated with MRI markers of vascular pathology in the absence of a significant decline in cognitive functions.

## Introduction

Hypertension is considered to play an important role in cognitive deficits, being mediated by microvascular dysfunction and cerebrovascular pathology (Iadecola, 2014). Cerebral small vessel disease has a constellation of clinical and radiological manifestations among which white matter hyperintensities (WMHs), lacunes, cerebral microbleeds and enlarged perivascular spaces have been associated with hypertension (Veglio et al., 2009; Brown et al., 2018). Advances in neuroimaging techniques with diffusion tensor imaging (DTI) have further improved our knowledge about the effects of hypertension on brain microstructure (Maillard et al., 2014; de Groot et al., 2015; Badji et al., 2019). For instance, high blood pressure was found to be associated with reduced fractional anisotropy (FA) in numerous white matter tracts in adults aged 46–100 years (mean 63.8) (de Groot et al., 2015). Interestingly, DTI metrics can capture microstructural changes related to hypertension before the appearance of irreversible white matter damage. Indeed, these metrics can capture microstructural changes within WMHs that are not yet visible on standard T2 fluid attenuation inversion recovery (FLAIR) images (Maillard et al., 2014).

Several studies have supported the vascular hypothesis of Alzheimer’s disease (VHAD) throughout the years. It is now more and more recognized that the critical steps that turn normal aging into a cognitively dysfunctional vortex starts with the acquisition of vascular risk factors such as hypertension (Baumgart et al., 2015; de la Torre, 2018). Vascular risk factors are responsible for the sustained cerebral hypoperfusion that follows (Baumgart et al., 2015; de la Torre, 2018). Mechanistically, once reached a critical threshold of cerebral hypoperfusion, hemodynamic deterioration will damage endothelial cells, thereby cerebral blood flow reactivity, vessel tone and vessel compliance as well as affecting the signaling pathways between astrocytes and neurons which in turns, ultimately lead to decline in brain metabolism and cognitive function (de la Torre, 2018).

Midlife hypertension is particularly associated with an increased risk of developing both Alzheimer’s disease (AD) and vascular dementia. Neuropathological studies showed that individuals with high blood pressure often have large areas of white matter hyperintensity, ventricular enlargement and silent infarcts, which can lead to cognitive dysfunction. A large autopsy-based neuropathological study importantly revealed that 80% of patients diagnosed with AD and no evidence of mixed dementia has vascular pathology including cortical infarcts, lacunes, and cerebral microbleeds (Toledo et al., 2013), supporting the concept that cerebrovascular dysfunction is prominent in AD and lowers the threshold for dementia for a given AD pathology burden (de la Torre, 2018).

Moreover, hypertension increases pulsatile aortic stress which promotes elastin fragmentation (Mitchell, 2014). These structural arterial changes successively lead to functional changes in CBF that have been associated with the rate of accumulation of cerebral AB over time (Hughes et al., 2014; Avolio et al., 2018). This means that overall there is a significant overlap between hypertension, cerebrovascular lesions and cerebral AB pathologies.

Because hypertension is a modifiable risk factor, blood pressure control has become an important candidate for the prevention of cerebrovascular pathology and cognitive impairment (Gorelick et al., 2017). Although several studies have assessed the impact of hypertension on markers of cerebral small vessel disease, white matter integrity, AD pathology or cognitive function, none have yet compared these markers between normotensive vs. different hypertensive groups (controlled, uncontrolled, and untreated hypertensives) in a single cohort of older adults. The aim of the present work is to study the clinical, MRI and CSF differences between normotensive, controlled hypertensive, uncontrolled hypertensive and untreated hypertensive older individuals aged 70 years old. In particular, we hypothesized that uncontrolled and untreated hypertensive participants have more vascular pathology, AD pathology and poorer cognitive performance than normotensive and hypertensive controlled participants.

## Materials and Methods

### Study Participants

As part of the Gothenburg H70 Birth Cohort Studies, 1,203 participants (559 men and 644 women, mean age 70.5 years) born in 1944 and registered as residents in Gothenburg agreed to participate (Rydberg Sterner et al., 2019). This means that all patients were assessed at ∼70 years of age. A previously published paper by Rydberg Sterner et al. (2019) contains all procedures for the baseline examination of the Birth cohort 1944, conducted in 2014–2016, represented in several key flow charts. For the current study, participants were excluded if they had a dementia diagnosis or mini-mental state examination score < 24 (*n* = 37), other neurological diseases (e.g., Parkinson and multiple sclerosis) (*n* = 15), or missing data in MRI or neuropsychological tests (*n* = 628). This left 523 individuals for the present study. However, only a subset number of these participants (*n* = 322) underwent CSF sampling via lumbar puncture. After removing participants according to our exclusion criteria stated above and the ones with missing variables, a total of 280 participants were included in all CSF analysis.

A general health interview was performed by a research nurse or a medical doctor, and consisted of questions about medical history, including questions regarding hypertension, diabetes, stroke, cardiac and neurological diseases, and present treatment. The interviews also contained questions regarding educational level, alcohol consumption and smoking. Physical examinations were carried out by research nurses and included measurement of blood pressure, anthropometry (i.e., height and weight), and an ECG. Systolic and diastolic blood pressure (SBP and DBP, respectively) were recorded in the right arm in the sitting position after 5 min rest using a standard cuff (Umedico) (Rydberg Sterner et al., 2019). The 12-lead ECG was coded according to the Minnesota Code (MC) by a biomedical analyst working at the cardiac laboratory at Sahlgrenska University Hospital.

Ethical approval was obtained from the Regional Ethical Review Board in Gothenburg (Approval Numbers: 869-13, T076-14, T166-14, 976-13, 127-14, T936-15, 006-14, T703-14, 006-14, T201-17, T915-14, 959-15, T139-15), and by the Radiation Protection Committee (Approval Number: 13-64) in accordance with the 1964 Helsinki declaration and its later amendment. Study participants provided written informed consent prior to examinations.

### Hypertension Classification

Participants were classified into four groups:

1) Normotensive (NT), *n* = 181.
i) No history of hypertension and normal blood pressure with SBP <140 mmHg and DBP <90 mmHG at the examination (*n* = 102).
ii) Previous history of hypertension but not taking antihypertensive medication and SBP <140 mmHg and DBP <90 mmHg at the examination (*n* = 79).
2) Hypertensive controlled (HTC), *n* = 46.
  History of hypertension, currently on anti-hypertensive treatment and SBP <140 mmHg and DBP <90 mmHg at the examination.
3) Hypertensive treated uncontrolled (HTU), *n* = 63.
  History of hypertension, currently on antihypertensive medication and SBP ≥140 mmHg or DBP ≥90 mmHg at the examination.
4) Hypertensive untreated (HU), *n* = 233.
i) History of hypertension, not taking antihypertensive medication and SBP ≥140 mmHg or DBP ≥90 mmHg at the examination (*n* = 90).
ii) No history of hypertension and either SBP ≥140 mmHg or DBP ≥90 mmHg at the examination (*n* = 143).

### Blood Sampling

Blood samples were drawn by a research nurse after an overnight fast. Level of total serum cholesterol, LDL, HDL and blood glucose were analyzed at the laboratory of the Department of Clinical Chemistry at Sahlgrenska University Hospital in Gothenburg with the use of standard routine clinical laboratory procedures as fully described by Rydberg Sterner et al. (2019). DNA was extracted from individual samples of whole blood according to standard procedures at LGC Genomics in Berlin (Germany). All the DNA samples have been genotypes at the University College London (UK) using the Neuro Consortium Array (neurox2) from Illumina^1^ as described in previous publications (Rydberg Sterner et al., 2019; Najar et al., 2021).

### Neurocognitive Assessments

Cognitive examination was performed by a psychiatric research nurse, a psychiatrist, or a medical doctor under supervision of a neuropsychologist. Participants underwent a series of tests covering a broad range of cognitive domains including memory (memory-in-reality + free recall, Thurston’s picture memory, ADAS-Cog word memory list, and delayed recall), attention and processing speed (figure identification, digit span forward, and supra-span test), executive function (digit span backward and SRBII – figure logic), verbal fluency (category fluency and letter fluency), and visuospatial abilities (Koh’s Block test). A detailed description of all cognitive tests and their respective scoring can be found in Table 1.

**TABLE 1:** Description of neuropsychological tests.

	Description	Score
**Memory**		
Word List, free recall	The participants are asked to remember 12 objects shown five minutes previously	Total number of objects the participants remembered [0–10]
Thurstone’s picture memory	Participants are shown 28 pictures and are then asked to recognize the correct picture among 3 others	Total number of pictures the participants recognized [0–28]
Memory-in-reality, delayed recall	Participants are shown objects and are prompted to recall the items in any order after 30 min	Total number of words the participants remembered [0–10]
**Attention/processing speed**		
Figure identification	The participants are shown similar one figure and five additional ones and should mark the identical figures among four others	Total number of images the participants correctly identified [0–60]
Digit span forward	Participants hear a sequence of numerical digits and are asked to recall the sequence correctly	Total number of “numbers” the participants were able to say [0–9]
Supra-span test	Participants hear a 10-words list related to clothing and are asked to recall as many words as possible	Total number of words the participants were able to remember [0–10]
**Executive function**		
Digit span backward	Participants hear a sequence of numerical digits and are asked to recall the sequence backward	Total number of “numbers” the participants were able to say [0–10]
Figure logic	Participants are shown five figures and need to say which one is different from the four others	Total number of images the participants correctly identified [0–30]
**Verbal fluency**		
Category fluency	Participants should name as many animals as possible within 1 min	Total number of animals the participants were able to say [0-]
Letter fluency	Participants should produce as many words as possible that begin with the letters F, A, and S within 1 min	Total number of object’s name the participants were able to say [0-]
**Visuospatial abilities**		
Koh’s block test	The participants are asked to assemble 4–16 blocks so that their upper surfaces replicate pattern as shown in seven subsequent figures	Total number of pattern the participant were able to reproduce [0–42]

### Cerebrospinal Fluid Sampling

All CSF samples were collected through lumbar puncture by a consultant neurologist/psychiatrist at the Neuropsychiatric outpatient department at the Sahlgrenska University Hospital. 10 ml of CSF was collected in a polypropylene tube following centrifugation at 1800 RCF (Relative Centrifugal Force) at 20°C for 10 min and stored at −70°C pending experimental use. CSF phosphorylated tau at threonine 181 (P-TAU) was determined using a sandwich enzyme-linked immunosorbent assay (ELISA) [INNOTEST^®^ htau Ag and PHOSPHO_TAU (P-TAU)], (Fujirebio, Ghent Belgium), as previously described (Blennow et al., 1995; Vanmechelen et al., 2000). CSF amyloid-β42 (Aβ42) was measured using a sandwich ELISA (INNOTEST^®^ β-amyloid 1–42), specifically constructed to measure amyloid-β peptides starting at amino acid 1 and ending at amino acid 42 (Sjögren et al., 2000). CSF Neurofilament light protein (NFL) was determined using a sandwich ELISA (UmanDiagnostics, Umeå, Sweden). The limit of detection for NFL was 125 ng/L. Laboratory procedures were accredited by the Swedish Board for Accreditation and Conformity and performed by board-certified laboratory technicians blinded to clinical data.

### Brain Magnetic Resonance Imaging Analysis

#### Magnetic Resonance Imaging Acquisition

All participants were scanned on a 3.0T Philips Achieva system (Philips Medical Systems) at the Aleris Clinic in Gothenburg. The imaging protocol included a 3D T1-weighted Turbo Field Echo (TFE) sequence to assess structural changes, a FLAIR sequence for detection of cerebral small vessel disease (e.g., white matter intensities and lacunes), a DTI sequence to assess white matter microstructural integrity, a venus bold sequence (VenoBOLD) for detection of microbleeds and a T2 weighted sequence for detection of enlarged perivascular spaces.

The T1w acquisition was done with sagittal slices of 1.0 mm isotropic resolution, Echo Time (TE) = 3.2 ms, Repetition Time (TR) = 7.2 ms and field of view (FOV) = 256 mm × 256 mm, flip angle = 9°, number of slices = 160. The FLAIR acquisition was done with sagittal slices of 2.0 mm isotropic resolution, TE = 280 ms, TR = 4,800 ms and FOV = 250 mm × 250 mm, flip angle = 90°, number of slices = 140. DTI data were acquired with axial slices of 3.0 mm isotropic resolution, encoded with 1 *b*-value shell: 800 k s/mm^2^, along with 32 directions and 1 b = 0 image. Other acquisition parameters were: TE = 83 ms, TR = 7,340 ms and FOV = 224 mm × 224 mm, flip angle = 90°, 32 volumes. The venus bold acquisition was done with 300 slices of 1.0 mm isotropic resolution, TE = 20,59–24,99 ms, TR = 14,59–17,60 ms, and FOV = 220 mm × 220 mm and finally, the T2w acquisition was done with transversal slices of 4.0 mm isotropic resolution, TE = 80 ms, TR = 3,000 ms and FOV = 230 mm × 230 mm.

#### Magnetic Resonance Imaging Data Processing

Magnetic resonance imaging data management and processing were done with our database system, the HiveDB (Muehlboeck et al., 2014).

##### T1w Processing

T1-weighted images underwent pre-processing with the Freesurfer pipeline (version 6.0.0) which include several steps described in the following link^2^ including correction of motion artifacts and spatial distortions, removal of non-brain tissue, alignment to the Talairach standard space, field intensity normalization, skull stripping, surface alignment, segmentation of subcortical white matter and deep gray matter structures, and cortical thickness estimation as the shortest distance between each vertex on gray matter/white matter surface and the gray matter/CSF (pial) surface (Fischl and Dale, 2000). The Freesurfer output underwent manual visual QC to ensure optimal estimation of thickness and volumes. For this study, the right and left hippocampal volumes were averaged and then adjusted for total intracranial volume. In addition, we calculated a meta temporal ROI as the mean cortical thickness of the entorhinal, inferior temporal, middle temporal and fusiform gyri (Jack et al., 2017).

##### Diffusion Tensor Imaging Processing

Diffusion-weighted images were analyzed using the FMRIB’s Diffusion Toolbox from FSL (Behrens et al., 2007). First, the data was corrected for distortions caused by eddy currents and head motion using the b0 non-diffusion data as a reference volume (Andersson and Skare, 2002). The resulting images were skull-striped and a diffusion tensor model was fitted at each voxel to obtain FA maps for each subject (Beaulieu and Allen, 1994). The FA maps were then submitted to a Tract-Based Spatial Statistics (Smith et al., 2007) analysis, which aligned these maps to standard space using non-linear registrations and merged them into a single 4D file. The mean of all FA images was fed into a skeletonization procedure to obtain a mean FA skeleton, which was thresholded with a FA value of 0.2. This skeleton represents the centers of all white matter tracts common to the group. Finally, all FA images were projected onto the thresholded mean FA skeleton, assigning the maximum FA value of the FA images to the skeleton voxel, and obtaining an image that contains the projected skeletonized FA data. Then, the mean FA values for each participant was computed within the FA skeleton mask and used in our analyses. As such the FA measure in the white matter included in our study is the mean FA of all voxels included in the FA skeleton and can be considered as a measure of global FA. The mean FA in the cingulum was also obtained directly from the ICBM-DTI-81 atlas (Mori et al., 2008) containing the right and left ventral aspect of the cingulum, referred to as the parahippocampal cingulum bundle.

##### Fluid Attenuation Inversion Recovery, T2w, and Venus Bold Processing

All MRI images were analyzed according to the recently proposed Standards for Reporting Vascular Changes on Neuroimaging and standardized scales (Gregoire et al., 2009; Wardlaw et al., 2013) by a trained radiologist (S.S). WMHs were classified from 0 to 3 (none or single punctate; multiple punctate; early confluent; and large confluent), according to the Fazekas scale (Fazekas et al., 1991). Lacunes were defined as having CSF signal on FLAIR (hypointense), T2 (hypertensive), and T1 (hypotensive), and be 3–15 mm in size. Cerebral microbleeds were rated according to the microbleed anatomical rating scales (MARS) and were defined as round hypointense dots on the venoBOLD acquisition. Microbleeds were only rated as present or absent (Gregoire et al., 2009). Care was taken to avoid cerebral microbleeds mimics (e.g., mineralization in the globus pallidus, hemorrhage, partial volume artifact etc.). Enlarged perivascular spaces were rated on T2 sequences in the centrum semiovale and basal ganglia as follows: 0 = none, 1 = 1–10, 2 = 11–20, 3 = 21–40, and 4 =>40, according to the validated enlarged perivascular space rating scale (MacLullich et al., 2004; Doubal et al., 2010; Potter et al., 2015).

### Statistical Analysis

To detect potential outliers, we visualized the histograms of all variables and computed their mean and median. Using the 1.5 interquartile rule, 26 outlier participants were excluded from all CSF analysis. To assess differences in demographics, clinical, MRI, neuropsychological and CSF variables between our four groups [(i) normotensive, (ii) hypertensive controlled, (iii) hypertensive uncontrolled, and (iv) hypertensive untreated], the Wilcoxon test was used for non-normally distributed variables. Chi-squared tests were used for binary variables.

Prior to analysis, the hippocampal volume where detrended for sex and intracranial volume, the neuropsychological variables were detrended for sex and education and the CSF variables were detrended for sex as done previously (Voevodskaya et al., 2014; Falahati et al., 2016). The detrending algorithm fits a generalized linear model (GLM) to each cognitive (CSF or MRI) variable in the control group (normotensive) to measure the effects of the covariates (education, sex, or intracranial volume) on the outcome in the absence of the group effect. This allows us to model the covariates-related changes as linear drift. Then, the regression coefficient of the resulting GLM model (linear drift) is used to remove the covariates-related changes from all participants and obtain corrected values. In order to do that, the detrended variable was first computed by summing the residual of the model with the mean of the prediction. Finally, a linear regression was performed on the detrended outcome over the group variable using the whole dataset. Age was not covaried for any of the variables since all subjects were assessed at around 70 years old. Before running the detrending algorithm, a preliminary GLM was performed to determine which covariates should be included. The sex was not found to predict hippocampal volume (*p* = 0.439) in our cohort and was therefore not included as covariate in the algorithm. In contrast, sex predicted 7 out of 12 specific tests for different cognitive functions and was therefore added to the detrending algorithm (*p* < 0.05). All statistical tests were implemented in R version 3.5.2. Correction for multiple comparisons was performed using the false discovery rate (FDR) procedure as described in Benjamini and Hochberg (1995) in SPSS (IBM SPSS 25 Statistics, Chicago, IL, United States). Since the *p*-threshold adjustment for significance under the Benjamini–Hochberg procedure not only depends on the number of tests but also on the calculated *p*-value for each test, original/uncorrected *p*-values were reported and only the significant ones after correction were highlighted in bold. The adjusted threshold under which a *p*-value is declared significant after the FDR correction is shown in caption legend. The FDR correction was based on the number of cognitive assessments within each cognitive domain, and the number of MRI metrics within each type of markers/sequences.

## Results

Table 2 shows the demographic and clinical characteristics of participants of the four groups (NC, HTC, HTU, and UT). As expected, SBP and DBP differed between the groups (Table 2). However, no significant differences were found in disease duration between the HTC and HTU groups. In general HTC, HTU, and HU had higher BMI and lower HDL cholesterol than NT. Interestingly HTC was found to have lower cholesterol than NT which could be explained by the fact that 43.5% of participants in HTC were on anti-cholesterol medication.

**TABLE 2:** Demographic and clinical characteristics.

Characteristic	Normotensive NT (*n* = 181)	Hypertensive treated (*n* = 115) Controlled HTC uncontrolled HTU (*n* = 46) (*n* = 63)		Hypertensive untreated HU (*n* = 233)	*P*-value
Female	94 [51.93%]	18 [39.13%]	26 [41.26%]	126 [54.07%]	/
Age (y)	70.54 ± 0.27	70.54 ± 0.231	70.51 ± 0.266	70.53 ± 0.245	/
Education (elementary school)	170 [93.92%]	41 [89.13%]	52 [82.53%]	209 [89.69%]	NT vs. HTU (*p* = 0.01383)
SBP (mmHg)	124.34 ± 8.63	125.56 ± 8.13	152.03 ± 14.52	152.34 ± 14.68	**NT vs. HTU (*p* = 2.2e-16).** **NT vs. HU (*p* = 2.2e-16).** **HTC vs. HTU (*p* = 2.2e-16).** **HTC vs. HU (*p* = 2.2e-16).**
DBP (mmHg)	74.01 ± 6.99	74.54 ± 5.91	84.28 ± 10.52	83.467 ± 8.019	**NT vs. HTU (*p* = 7.34e-16).** **NT vs. HU (*p* = 2.2e-16).** **HTC vs. HTU (*p* = 3.82e-16).** **HTC vs. HU (*p* = 2.31e-16),**
Hypertension DD (y)	0	13.85 ± 10.57	11.66 ± 10.37	NA	/
**APOE**-ε**4 status**					**/**
One allele	55 [30.38%]	16 [34.78%]	13 [20.63%]	71 [30.47%]	**/**
Two alleles	2 [1.10%]	2 [4.34%]	6 [9.52%]	6 [2.57%]	**NT vs. HTU (*p* = 4.36e-4).** HTU vs. HU (*p* = 0.023)
**Vascular risk factors**					
Diabetes	18 [9.94%]	6 [13.04%]	14 [22.22%]	27 [11.58%]	NT vs. HTU (*p* = 0.023). HTU vs. HU (*p* = 0.050).
Current smoking	18 [9.94%]	5 [10.86%]	2 [3.17%]	12 [5.15%]	/
Alcohol risk consumption (grams per week)	93.41 ± 92.31	104.39 ± 100.48	72.85 ± 69.71	87.82 ± 96.16	/
BMI (km/m^2^)	24.84 ± 3.87	26.74 ± 3.26	28.17 ± 4.75	26.01 ± 4.20	**NT vs. HTC (*p* = 3.92e-5).** **NT vs. HTU (*p* = 4.42e-07).** **NT vs. HU (*p* = 2.94e-4).** **HTU vs. HU (*p* = 1.02e-4).**
**Medical conditions**					
Ischemic heart diseases	19 [10.49%]	7 [15.21%]	9 [14.28%]	14 [6.00%]	/
Heart failure	2 [1.10%]	2 [4.34%]	2 [3.17%]	2 [0.85%]	/
Atrial fibrillation	14 [7.73%]	7 [15.21%]	3 [4.76%]	13 [5.57%]	HTU vs. HU (*p* = 0.030)
Stroke					
**Lipid profile**					
Tot cholesterol, mmol/L	5.57 ± 1.18	4.99 ± 1.25	5.23 ± 1.05	5.68 ± 1.20	**NT vs. HTC (*p* = 3.70e-4).** NT vs. HTU (*p* = 0.035). **HTC vs. HTU (*p* = 7.50e-5).** **HTU vs. HU (*p* = 6.840e-4).**
LDL cholesterol, mmol/L	3.53 ± 1.06	3.16 ± 1.05	3.27 ± 0.92	3.63 ± 1.04	NT vs. HTC (*p* = 0.039). **HTC vs. HU (*p* = 8.39e-4).** **HTU vs. HU (*p* = 0.015).**
HDL cholesterol, mmol/L	1.74 ± 0.55	1.50 ± 0.43	1.51 ± 0.40	1.76 ± 0.52	**NT vs. HTC (*p* = 6.89e-4).** **NT vs. HTU (*p* = 3.38e-4).** **HTC vs. HTU (*p* = 1.80e-4).** **HTU vs. HU (*p* = 3.01e-5).**
Anti-cholesterol medication, number, and (%)	33 (18.2%)	20 (43.5%)	34 (53.96%)	39 (16.7%)	**NT vs. HTC (*p* = 6.28e-4).** **NT vs. HTU (*p* = 1.097e-7).** **HTC vs. HU (*p* = 1.129e-4).** **HTU vs. HU (*p* = 3.265e-09).**

*Data are presented as means ± standard deviations or number [proportion%]. y, years; SBP, systolic blood pressure; DBP, diastolic blood pressure; DD, disease duration; APOE-ε4, apolipoprotein ε4 allele; Tot, total; LDL, low-density lipoprotein; HDL, high-density lipoprotein. Ischemic heart diseases are defined by either myocardial infarction or angina pectoris. Information about diabetes was ascertained based on self-report medical history, use of hypoglycemic medications (diet, oral hypoglycemic agents, or insulin), or fasting blood glucose ≥ 7.0 mmol/L or non-fasting blood glucose (noFBG) ≥ 11 mmol/L as suggested by the American Diabetes Association’s (American Diabetes Association, 2019). Only results with uncorrected p < 0.05 are shown. Significant results after FDR are in bold. Adjusted threshold with FDR are p = 0.008 for education, APOE-ε4, diabetes and atrial fibrillation, p = 0.033 for SBP, DBP, BMI, and HDL cholesterol, p = 0.016 for LDL cholesterol and p = 0.025 for total cholesterol.*

### Normotensive Versus Hypertensive Participants

#### Magnetic Resonance Imaging Markers of Alzheimer’s Disease, Neurodegeneration and Vascular Pathology

Hypertensive participants were found to have more white matter pathology than NT (Table 3). For instance, HTC had more lacunes (*p* = 4.45e-4) than NT. HTU had more lacunes than NT, but also lower FA (*p* = 3.08e-4). HTU had more WMHs and epvsCS than NT (*p* = 1.29e-4 and *p* = 0.026, respectively), however, the latter was not significant after FDR corrections. In contrast, no differences were found between NT and HU regarding any MRI markers. HTU had more lacunes (*p* = 3.03e-4), more WMHs (*p* = 0.022) than HU. HTU was also found to have lower FA (*p* = 0.02) and more microbleeds (*p* = 0.037) than HU, although these results were not significant after FDR.

**TABLE 3:** Differences in MRI variables among normotensive and hypertensive participants (treated controlled, uncontrolled, or untreated).

	Normotensive NT (*n* = 181)	Hypertensive treated (*n* = 115) Controlled HTC uncontrolled HTU (*n* = 46) (*n* = 63)		Hypertensive untreated HU (*n* = 233)	*P*-value
**Gray matter**					
Temporal thickness	2.91 ± 0.14	2.89 ± 0.13	2.92 ± 0.14	2.92 ± 0.12	/
Hippocampal volume	3951.373 ± 384.478	3792.429 ± 431.198	3926.912 ± 454.671	3898.523 ± 417.834	NT vs. HTC (*p* = 0.0308). HTC vs. HU (*p* = 0.0405).
**Microstructure**					
FA cingulum	0.23 ± 0.05	0.22 ± 0.02	0.23 ± 0.02	0.23 ± 0.05	/
FA white matter	0.40 ± 0.06	0.39 ± 0.06	0.39 ± 0.05	0.40 ± 0.05	**NT vs. HTU (*p* = 3.08e-4).** HTU vs. HU (*p* = 0.020).
**cSVD markers**					
Microbleed	0.24 ± 1.04 [0–12]	0.24 ± 0.97 [0–6]	0.81 ± 3.04 [0–22]	0.18 ± 0.83 [0–8]	HTU vs. HU (*p* = 0.037)
WMHs	0.99 ± 0.57 [0–3]	1.06 ± 0.71 [0–3]	1.30 ± 0.73 [0–3]	1.08 ± 0.63 [0–3]	**NT vs. HTU (*p* = 1.29e-4).** HTU vs. HU (*p* = 0.022).
epvsCS	1.95 ± 0.74 [1–4]	NA	2.21 ± 0.81 [1–4]	1.97 ± 0.82 [0–4]	NT vs. HTU (*p* = 0.026).
epvsBG	1.24 ± 0.52 [1–3]	NA	1.35 ± 0.63 [1–4]	1.25 ± 0.56 [0–3]	**/**
Lacunes	0.05 ± 0.28 [0–3]	0.17 ± 0.44 [0–2]	0.25 ± 0.59 [0–2]	0.073 ± 0.32 [0–3]	**NT vs. HTC (*p* = 4.45e-4).** **NT vs. HTU (*p* = 3.35e-5).** HTC vs. HU (*p* = 0.03). **HTU vs. HU (*p* = 3.02e-4).**

*Data are presented as means ± standard deviations [range] is added for non-continuous variables. FA, fractional anisotropy; cSVD, cerebral small vessel disease; WMHs, white matter hyperintensities; epvs, enlarged perivascular space; CS, centrum semiovale; BG, basal ganglia. Only results with uncorrected p < 0.05 are shown. Significant results after FDR are in bold. Adjusted threshold with FDR are p = 0.008 for white matter microstructure and cerebral microbleeds and p = 0.011 for all cSVD markers.*

No significant differences were found in MRI markers of AD/neurodegeneration pathology between normotensives and the hypertensive groups.

#### Cerebrospinal Fluid Markers of Alzheimer’s Disease Pathology and Neurodegeneration

Hypertensives groups had comparable levels of P-TAU, NFL, and Aβ42, compared to NT (Table 4), and their were no differences in any CSF variables between the hypertensive groups.

**TABLE 4:** Differences in CSF variables among normotensive and hypertensive participants (treated controlled, uncontrolled, or untreated).

	Normotensive NT (*n* = 81)	Hypertensive treated (*n* = 60) Controlled HTC uncontrolled HTU (*n* = 22) (*n* = 38)		Hypertensive untreated HU (*n* = 113)	*P*-value
**P-tau**	44.41 ± 12.02	48.95 ± 15.24	44.37 ± 12.07	47.80 ± 12.48	/
**NFL**	723.69 ± 270.94	702.38 ± 224.34	724.70 ± 271.42	728.20 ± 219.70	/
**AB42**	717.87 ± 280.68	721.84 ± 229.71	731.19 ± 281.78	726.39 ± 226.15	/

*Data are presented as means ± standard. P-TAU, phosphorylated TAU; NFL, neurofibril, AB42. Only results with uncorrected p < 0.05 are shown. Significant results after FDR are in bold.*

#### Cognition

The cognitive performance between NT and hypertensive participants was in general comparable (Table 5). However, HTC performed significantly worse than HU in MMSE (Table 5, *p* = 0.008). Interestingly, HTC was also found to perform worse than HU in the Supra-span test (Table 5, *p* = 0.012) and Koh’s Block test (Table 5, *p* = 0.011), which assess attention/processing speed and visuospatial performance, respectively. However, the former results were not significant after FDR correction. In addition, HTU participants had lower performance than HU in delay recall memory test (*p* = 0.047), although this result was not significant after FDR correction.

**TABLE 5:** Differences in cognitive performances among normotensive and hypertensive participants (treated controlled, uncontrolled, or untreated).

	Normotensive NT (*n* = 181)	Hypertensive treated (*n* = 115) Controlled HTC uncontrolled HTU (*n* = 46) (*n* = 63)		Hypertensive untreated HU (*n* = 233)	*P*-value
**General cognition**					
MMSE score	29.16 ± 1.14	28.62 ± 1.31	29.16 ± 1.17	29.14 ± 1.06	**NT vs. HTC (*p* = 0.001).** **HTC vs. HTU (*p* = 0.013).** **HTC vs. HU (*p* = 0.008).**
**Memory**					
Word list and free recall	8.44 ± 1.51	8.30 ± 2.00	8.51 ± 1.62	8.70 ± 1.39	/
Thurstone’s picture memory	21.60 ± 4.34	21.92 ± 4.73	22.57 ± 3.79	22.59 ± 3.27	/
Memory-in-reality and delay recall	7.41 ± 1.77	7.54 ± 1.71	7.20 ± 1.81	7.61 ± 1.89	HTU vs. HU (*p* = 0.047)
**Attention/processing speed**					
Figure identification	28.82 ± 8.06	27.15 ± 7.21	26.84 ± 7.94	28.07 ± 7.60	/
Digit span forward	6.05 ± 1.13	5.92 ± 1.16	5.75 ± 1.09	5.98 ± 1.15	**/**
Supra-span test	7.54 ± 1.48	7.28 ± 1.21	7.52 ± 1.44	7.69 ± 1.36	HTC vs. HU (*p* = 0.012)
**Executive function**					
Digit span backward	4.43 ± 1.13	4.19 ± 0.87	4.38 ± 1.23	4.40 ± 1.22	/
Figure logic	20.23 ± 4.16	19.54 ± 4.23	19.87 ± 4.50	20.07 ± 3.98	**/**
**Verbal fluency**					
Category fluency	24.45 ± 6.01	24.20 ± 6.13	24.65 ± 7.55	23.93 ± 5.55	/
Letter fluency	40.99 ± 13.32	40.65 ± 14.93	38.23 ± 12.95	40.27 ± 11.99	/
**Visuospatial abilities**					
Koh’s block test	7.45 ± 2.17	7.04 ± 1.85	7.59 ± 2.34	7.80 ± 2.18	HTC vs. HU (*p* = 0.011)

*Data are presented as means ± standard deviations. Only results with uncorrected p < 0.05 are shown. Significant results after FDR are in bold. Adjusted threshold with FDR is p = 0.025 for general cognition, p = 0.002 for memory and attention/processing speed, and p = 0.008 for visuospatial abilities.*

## Discussion

We examined the clinical, neurochemical and radiological differences between normotensives and those with controlled, uncontrolled or untreated hypertension, using MRI markers of vascular pathology (WMHs, lacunes, microbleeds, enlarged perivascular space, and FA), MRI and CSF markers of AD pathology/neurodegeneration, as well as cognitive tests in a cognitively healthy population-based sample of 70-year-olds.

Firstly, we observed a higher vascular burden in uncontrolled hypertensive participants compared to the other groups. Secondly, participants with treated but uncontrolled hypertension had more vascular pathology than those with untreated hypertension. This suggests that treatment of hypertension may not be enough to prevent further cerebrovascular pathology, good control of blood pressure may also be necessary. Thirdly, CSF and MRI markers of AD pathology/neurodegeneration did not differ between normotensive and hypertensive participants, nor between hypertensive subgroups, suggesting that hypertension may be associated with cognitive changes in late life through cerebrovascular pathology rather than AD related pathology. Finally, in this sample of dementia-free participants, cognitive function did not differ between normotensive and hypertensive participants, nor between the hypertensive groups, suggesting that MRI markers of vascular pathology can capture cerebrovascular changes that have not yet translated into cognitive symptoms.

### Pathogenesis of Hypertensive Brain Damage

The negative effects of hypertension on cognitive function are best understood in terms of the brain’s need for a certain constant perfusion threshold for optimal function. Indeed, the brain is a highly vascularized organ, making continuous regulation of blood flow critical to meet its high metabolic demand (Cipolla, 2009). A high blood pressure can alter the structure and molecular composition of cerebral blood vessels, which ultimately compromises oxygen and glucose delivery for proper neural function as well as removal of metabolic waste and toxin protein such as amyloid-β plaques and P-TAU (Iadecola, 2010; Pires et al., 2013). These alterations render the brain more vulnerable to ischemic injury, small vessel disease, and the development of neurodegenerative pathologies, such as AD (Faraco and Iadecola, 2013; Sörös et al., 2013).

### Cerebrovascular Pathology

#### Normotensive vs. Hypertensive Treated Controlled

Cerebral WMHs are more common and extensive in patients with cardiovascular risk factors such as hypertension, diabetes mellitus and heart disease (Pantoni and Garcia, 1995; Skoog, 1998; Debette and Markus, 2010; Habes et al., 2018). WMHs have been characterized by different pathologies, including axonal and myelin loss, astrogliosis, reduction of oligodendrocytes, microglial activation and white matter infarction (Braffman et al., 1988; Fazekas et al., 1993). This explains why the different mechanisms contributing to the heterogeneous pathology within WMHs have not been completely elucidated yet (Joutel and Chabriat, 2017). Cerebral hypoxia due to stenosis or occlusion of vessels caused by hypertension is considered as the major contributor of WMHs (Iadecola, 2013; Badji et al., 2019). However, we found no differences in WMHs load between normotensives and treated controlled hypertensives. This suggests that at least some of the WMHs may reflect benign, age-associated changes, such as partial loss of the ependymal lining (Mito et al., 2019).

Elevated blood pressure is associated with microatheromatosis and atherosclerosis, giving rise to thickening of the vessel wall or, in severe cases, to vessel wall necrosis, which may lead to rupture (Alistair, 2002; Martí-Vilalta et al., 2011). These morphological alterations facilitate the appearance of lacunes, which were more common in participants with controlled hypertension compared to the normotensives as also found by others (Dozono et al., 1991; Kombate et al., 2012).

#### Normotensive vs. Hypertensive Treated Uncontrolled

Previous studies reported that hypertension is associated with reduced microstructural integrity in most white matter tracts, as assessed by DTI metrics in both younger and older adults (Maillard et al., 2012; Rosano et al., 2015). We found that uncontrolled hypertensive participants had more lacunes than normotensives, but also a lower FA, suggesting that uncontrolled blood pressure leads to further microstructural damage. A recent study assessed the microstructural integrity of 9 white matter tracts in a cohort of middle-aged adults with hypertension (age range 56,7–65.6 years) (McEvoy et al., 2015). In contrast to our findings, this study reported that white matter alterations were more often observed in both controlled and uncontrolled hypertensives compared to normotensives. However, this study only examined men, who may be more vulnerable to the effect of hypertension (Reckelhoff, 2001). The Third National Health and Nutrition Examination survey (NHANES III) reported that despite an increased prevalence of hypertension in middle-aged men compared to woman, men seems to receive less treatment for hypertension compared to woman (Burt et al., 1995; August, 1999). Thus, only 19% of men had their blood pressure controlled compared to 28% of women (McEvoy et al., 2015).

We even found that uncontrolled hypertensive participants had more WMHs and more enlarged perivascular spaces than properly controlled treated hypertensive participants. However, this result was not significant after corrections for multiple comparisons. Similar findings have been found in the literature. For instance, Kuller et al. (2010) found that woman above age 65 years, who were treated for hypertension, but had uncontrolled blood pressure, had the greatest amount of total WMHs volume and number of regions containing WMHs than women with controlled hypertension (with or without treatment). This suggests that uncontrolled blood pressure may trigger mechanisms responsible for the appearance of WMHs. For instance, increased blood brain barrier permeability in hypertension may lead to water shift and vasogenic edema (Farrall and Wardlaw, 2009; Maclullich et al., 2009). Fluid stagnation may further lead to enlargement and distortion of perivascular spaces (Brown et al., 2018), in line with our results. Future studies using more advanced diffusion models, such as the single-shell 3-Tissue CSD (SS3T-CSD) methodology, available in MRtrix3Tissue^3^, a fork of the MRtrix3 software framework), proposed by Dhollander and Connelly (2016) and Dhollander et al. (2017) may help us better understand the impact of hypertension on the micro-structural properties of normal-appearing WM as well as other tissues and fluids (free water). Our result further suggests that treatment of hypertension may not be enough to slow down WMHs progression and additional cerebrovascular pathology. Blood pressure may need to be efficiently controlled as well, for adequate cerebrovascular protection. Future studies may combine measures of blood pressure variability of 24 h and the SS3T-CSD methodology to better understand the impact of uncontrolled hypertension on white matter integrity.

#### Hypertensive Treated Uncontrolled vs. Hypertensive Untreated

Our finding of an overall better white matter integrity in untreated hypertensives compared with those treated but uncontrolled may seem contradictory. One explanation could be that misclassification occurred in the untreated group. Some of these participants did not receive a diagnosis of hypertension by a physician and were classified as “untreated hypertensive” based on a single measurement of office blood pressure. In some participants, blood pressure could have been elevated due to a white coat effect. Another possibility is that hypertension disease duration may be shorter in the untreated group. Consequently, the untreated hypertensive participants enrolled in this study may not exhibit the accompanying white matter changes because they are either not truly hypertensive or do not have significant white matter damage yet due to a shorter disease duration.

Nevertheless, such discrepancy is also found in other studies. For instance, Gons et al. (2012) reported a higher structural integrity of the corpus callosum in untreated hypertensive participants compared with treated controlled and uncontrolled individuals, which is in line with our findings. These results emphasize that both treatment and optimal control of blood pressure may be critical for efficient cerebrovascular protection.

### Magnetic Resonance Imaging and Cerebrospinal Fluid Biomarkers Reflecting Alzheimer’s Disease Pathophysiology and Neurodegeneration

Few studies have examined the association between hypertension and MRI manifestations of AD in non-clinical populations (Skoog and Gustafson, 2006). For instance, Lane et al. (2019) showed that increased SBP in middle age (age 36–43) was associated with smaller hippocampal volumes at age 69–71 years. In addition, in the Honolulu-Asia Study and the Rotterdam study, participants with untreated hypertension had an increased risk for hippocampal atrophy (Korf et al., 2004; den Heijer et al., 2005). However, we found no significant differences in hippocampal volumes between normotensive and hypertensive individuals (controlled, uncontrolled with medication or untreated). A recent study also showed no significant hippocampal atrophy in treated but uncontrolled hypertensive older adults compared to normotensive individuals (Wiseman et al., 2004), which is in agreement with our findings. Differences in MRI protocol, means of hippocampal delineation (manual vs. automatic) and hypertensive group characterization may explain these discrepancies. Another potential explanation is that hippocampal atrophy could be a later sign of cognitive decline. In contrast, white matter pathology may represent the underlying factor associated with cognitive decline when hypertension occurs. This hypothesis is strengthened by results of longitudinal studies showing that although long-term exposure to high blood pressure predicts the appearance of markers of cerebral small vessel disease, it does not predict hippocampal atrophy (Allan et al., 2015).

In addition, we did not find a relationship between hypertension and the pathological CSF hallmarks of AD. A few evidence exists to support that hypertension is associated to amyloid plaques and neurofibrillary tangles in the brain (Roberts et al., 2008; Gottesman et al., 2010; Shah et al., 2012; Lane et al., 2019), however, no consensus has yet been reached.

### Cognition

Hypertension has been associated with mild cognitive deficits in many studies (Longstreth et al., 1996; Harrington et al., 2000). However, most studies assessed cognitive performance in older adults by comparing normotensive individuals to untreated hypertensive individuals (Asmar et al., 1995; Harrington et al., 2000; Wysocki et al., 2012; Desjardins-Crépeau and Bherer, 2016). Although several reports suggest that the use of antihypertensive drugs may reduce the incidence of cognitive decline and AD (Skoog et al., 1996; Guo et al., 1999; Hajjar et al., 2005; Khachaturian et al., 2006), the positive impact of antihypertensive medications on the risk of cognitive decline and dementia has not yet reached a consensus. It is thus important to compare the cognitive performance of normotensive and different hypertensive groups (controlled, uncontrolled, and untreated) in older adults. Our study suggests that, in general, the cognitive performance of normotensive and hypertensive groups were similar in 70-year-olds which suggest that MRI markers of cerebral small vessel disease are able to identify white matter changes that do not yet translate into a decline in cognition. A recent study compared cognitive performance in controlled hypertensive and normotensive individuals aged 60–75 years, who were free from dementia (Noriega de la Colina et al., 2019). In that study, controlled hypertensives participants had a lower performance in immediate and delayed recall and total number of words in the Rey Auditory Verbal Learning test compared to normotensives. This is consistent with similar other findings in the literature (Swan et al., 1998; Fitri and Rambe, 2018). After dividing the hypertensive groups into controlled and uncontrolled hypertensive patients in individuals who received antihypertensive medication for at least 6 months, Spinelli et al. (2014) found that uncontrolled individuals performed worse in tests evaluating attention and executive function. One should consider the fact that such an effect may be due to the antihypertensive drugs themselves which can affect mood or attention, in particular when combined, or withdrawn abruptly, resulting in orthostatic hypotension and decline in cognitive function (Johansen et al., 2012; Moonen et al., 2016). However, results from the SPRINT-MIND study showed that intensive SBP control to reduce SBP to 120 mmHg in comparison to standard antihypertensive protocol aiming to control the SBP to 140 mmHg significantly reduced the rate of mild cognitive impairment by 19% (Sprint Mind Investigators for the Sprint Research Group et al., 2019). This means that not only treatment, but treatment protocol has an impact on cognitive function. Further studies are needed to demystify not only the relative contribution of treatment and treatment regimen but also should include other factors such as age and duration of hypertension.

### Strengths and Limitations

To our knowledge, this is the first large study investigating clinical, CSF, MRI, and cognitive differences between normotensive and controlled, uncontrolled and untreated hypertensive individuals in a population-based sample of 70-year-olds. A strength of our study is the use of several MRI markers (WMHs, lacunes, microbleeds, enlarged perivascular space, FA, hippocampal volume, and temporal thickness), which captures a broad range of AD-related cerebrovascular pathology. In addition, we used various neuropsychological tests (Table 1) sensitive to different cognitive functions (memory, attention/processing speed, executive function, verbal fluency, and visuospatial abilities). As mentioned above, a strength of this study is the inclusion of CSF measures, though relatedly, a limitation is that the sample size for the CSF analyses was much smaller.

An important limitation of this study is the use of static rather than 24-h ambulatory blood pressure measurements, which are more closely related to end-organ damage. Another limitation is the use of a single blood pressure measurement, which is less reliable than multiple measurements. Thus, the hypertensive untreated group contains 90 true untreated hypertensive participant (with a previous history of hypertension, but not taking antihypertensive drugs and with SBP ≥140 mmHg or DBP ≥90 mmHg), and 143 potential previously unknown hypertensive participants (no history of hypertension and either SBP ≥140 mmHg or DBP ≥90 mmHg). The reason to merge the two groups is that a single blood pressure is not a reliable estimate of hypertension disease therefore this sub-group cannot be considered as a reliable hypertensive unknown group. Moreover, as mentioned in the discussion, misclassification could have occurred in this group. A third limitation is the cross-sectional design, which limits conclusions regarding directions of associations. Comparing subgroups based on hippocampal atrophy, pathological results in cognition or CSF changes suggestive of AD would have been interesting, however, due the limited sample size in certain groups (i.e., 46 participants in HTC) and its associated statistical caveat, we refrained from doing such comparison. Nevertheless, we provided a Supplementary Table 1 presenting qualitative information on such comparisons. However, a follow-up study using a much larger sample size, will focus on the cerebrovascular differences between HTC and HTU using the SS3T-CSD methodology mentioned in the manuscript and will also include the comparisons mentioned above. Finally, differences in APOE4 have not been discussed considering the difficulty to draw any accurate conclusion from our results.

## Conclusion

This study is the first to provide a comprehensive investigation of the clinical, MRI and CSF differences between normotensive, controlled hypertensive, uncontrolled hypertensive and untreated hypertensive older individuals aged 70 years old. Using several different markers of cerebrovascular pathology, this work provides evidence that hypertensive participants controlled with medication have more subcortical infarcts, such as lacunes, than normotensives. Uncontrolled blood pressure despite medication seems to trigger additional mechanisms that translate into changes in the white matter microstructure, fluid stagnation and enlargement of perivascular spaces. These results stress that not only treatment, but good control of blood pressure is essential for efficient cerebrovascular protection. Considering the strong interplay between hypertension and decline in cerebrovascular integrity, the present work further emphasizes the need for novel interventions aiming to preserve the white matter and cerebrovascular integrity. Finally, our results show no differences in any MRI or CSF markers of AD and neurodegeneration between the normotensive and hypertensive groups. This suggests that the general hypertensive population, including the one that is uncontrolled despite medication, does not translate into differences in AD-related neuropathology in 70-year-olds.

## Data Availability Statement

The raw data supporting the conclusions of this article will be made available by the authors, without undue reservation, to any qualified researcher.

## Ethics Statement

The studies involving human participants were reviewed and approved by the Regional Ethical Review Board in Gothenburg (Approval Numbers: 869-13, T076-14, T166-14, 976-13, 127-14, T936-15, 006-14, T703-14, 006-14, T201-17, T915-14, 959-15, and T139-15) and by the Radiation Protection Committee (Approval Number: 13-64) in accordance with the 1964 Helsinki declaration and its later amendment. The participants provided their written informed consent to participate in this study.

## Author Contributions

AB: design of the study, analysis, statistics, writing, and revision of the article. JP, L-OW, and EW: design of the study, analysis, and revision of the article. SS and AM: analysis and revision of the article. JS and LR: collecting data, analysis, and revision of the article. KP: statistics and revision of the article. KB, HZ, SK, and AZ: collection of data and revision of the article. IS: design of the study and revision of the article. All authors contributed to the article and approved the submitted version.

## Conflict of Interest

KB has served as a consultant, at advisory boards, or at data monitoring committees for Abcam, Axon, Biogen, JOMDD/Shimadzu, Julius Clinical, Lilly, MagQu, Novartis, Roche Diagnostics, and Siemens Healthineers, and is a co-founder of Brain Biomarker Solutions in Gothenburg AB (BBS), which is a part of the GU Ventures Incubator Program, all unrelated to the present study. HZ has served at scientific advisory boards for Denali, Roche Diagnostics, Wave, Samumed, Siemens Healthineers, Pinteon Therapeutics and CogRx, has given lectures in symposia sponsored by Fujirebio, Alzecure and Biogen, and is a co-founder of Brain Biomarker Solutions in Gothenburg AB (BBS), which is a part of the GU Ventures Incubator Program (outside submitted work). SK has served at scientific advisory boards for Biogen and Geras Solutions. The remaining authors declare that the research was conducted in the absence of any commercial or financial relationships that could be construed as a potential conflict of interest.

## Publisher’s Note

All claims expressed in this article are solely those of the authors and do not necessarily represent those of their affiliated organizations, or those of the publisher, the editors and the reviewers. Any product that may be evaluated in this article, or claim that may be made by its manufacturer, is not guaranteed or endorsed by the publisher.

## Footnotes

1
www.illumina.com

2
http://surfer.nmr.mgh.harvard.edu/fswiki/FreeSurferAnalysisPipelineOverview

3
https://3Tissue.github.io
