# Supplementary material for: Cerebrospinal Fluid Biomarkers, Brain Structural and Cognitive Performances Between Normotensive and Hypertensive Controlled, Uncontrolled and Untreated 70-Year-Old Adults
Source: Front Aging Neurosci. 2022 Jan 12;13:777475. doi: 10.3389/fnagi.2021.777475 (PMC8791781; doi:10.3389/fnagi.2021.777475)
Supplement: Supplementary file 1 [file Table_1.docx]

|  | **Normotensive**  NT  (n=181) | **Hypertensive Treated** (n=115)    Controlled HTC uncontrolled HTU  (n=46) (n=63) | | **Hypertensive untreated**  HU  (n=233) |
| --- | --- | --- | --- | --- |
| **Hippocampal volume** |  |  |  |  |
| SD  (n) | <1.5 \| ≥ 1.5  (162) \| (19) | <1.5 \| ≥ 1.5  (37) \| (9) | <1.5 \| ≥ 1.5  (53) \| (10) | <1.5 \| ≥ 1.5  (201) \| (32) |
| mean | 3908.92 \| 4202.65 | 3863.76 \| 3439.97 | 3893.53 \| 3884.45 | 3946.33 \| 3749.11 |
| **MMSE** |  |  |  |  |
| SD  (n) | <1.5 \| ≥ 1.5  (165) \| (16) | <1.5 \| ≥ 1.5  (39) \| (7) | <1.5 \| ≥ 1.5  (54) \| (9) | <1.5 \| ≥ 1.5  (215) \| (18) |
| mean | 29.47 \| 26.25 | 29.07 \| 25.85 | 29.46 \| 26.77 | 29.38 \| 26.27 |
| **Koh’s Block test** |  |  |  |  |
| SD  (n) | <1.5 \| ≥ 1.5  (158) \| (23) | <1.5 \| ≥ 1.5  (40) \| (6) | <1.5 \| ≥ 1.5  (51) \| (12) | <1.5 \| ≥ 1.5  (199) \| (34) |
| mean | 7.65 \| 5.94 | 7.09 \| 8.05 | 7.50 \| 8.66 | 7.61 \| 8.51 |
| **Word List, free recall** |  |  |  |  |
| SD  (n) | <1.5 \| ≥ 1.5  (158) \| (23) | <1.5 \| ≥ 1.5  (40) \| (6) | <1.5 \| ≥ 1.5  (53) \| (10) | <1.5 \| ≥ 1.5  (217) \| (16) |
| mean | 8.92 \| 5.25 | 8.81 \| 4.29 | 9.02 \| 5.40 | 8.98 \| 5.26 |
| **Supra-span test** |  |  |  |  |
| SD  (n) | <1.5 \| ≥ 1.5  (142) \| (39) | <1.5 \| ≥ 1.5  (42) \| (4) | <1.5 \| ≥ 1.5  (54) \| (9) | <1.5 \| ≥ 1.5  (194) \| (39) |
| mean | 7.63 \| 7.35 | 7.27 \| 6.19 | 7.62 \| 6.34 | 7.67 \| 7.99 |
| ***Sample of the cohort that underwent CSF sampling*** | | |  |  |
|  | (n=81) | (n=22) | (n=38) | (n=113) |
| **AB42** |  |  |  |  |
| pg/mL  (n) | ≤ 530 \| >530  (25) \| (56) | ≤ 530 \| >530  (6) \| (16) | ≤ 530 \| >530  (11) \| (27) | ≤ 530 \| >530  (22) \| (91) |
| mean | 429.18 \| 846.75 | 448.66 \| 824.29 | 452.57 \| 844.70 | 469.65 \| 788.47 |
| **p-tau** |  |  |  |  |
| pg/mL  (n) | ≥ 80 \| <80  (0) \| (81) | ≥ 80 \| <80  (0) \| (22) | ≥ 80 \| <80  (0) \| (33) | ≥ 80 \| <80  (2) \| (111) |
| mean | (0) \| (44.41) | (0) \| (48.95) | (0) \| (44.37) | (81.46) \| (47.19) |

**Table SM1: Qualitative subgroups comparisons based on hippocampal atrophy, pathological results in cognition in selected cognitive tests and CSF changes suggesting of AD.** Abbreviations: P-TAU = phosphorylated TAU. The following CSF cut-offs were used to define AD biomarker pathology: CSF A42 levels ≤530 pg/mL (the A criterion), P-tau levels of ≥80 pg/mL (the T criterion).
